# *In vivo* antitumor effects of carboxymethyl chitosan-conjugated triptolide after oral administration

**DOI:** 10.1080/10717544.2020.1770370

**Published:** 2020-06-08

**Authors:** Huahui Zeng, Xin Zhu, Qikang Tian, Yinyin Yan, Lan Zhang, Min Yan, Ruiqin Li, Xiaofang Li, Guoqiang Wang, Jinlian Ma, Yufang Su, Xiangbo Zhang, Linyu Ma, Zhenqiang Zhang, Xiangxiang Wu

**Affiliations:** a Academy of Chinese Medicine Sciences, Henan University of Chinese Medicine, Zhengzhou, China; b School of Basic Medicine, Henan University of Chinese Medicine, Zhengzhou, China; c Pharmacy College, Henan University of Chinese Medicine, Zhengzhou, China

**Keywords:** Triptolide, carboxymethyl chitosan, oral administration, pancreatic cancer, toxicity

## Abstract

The purpose of this study is to evaluate *in vitro and in vivo* antitumor efficacy and subacute toxicity of triptolide (TP) prodrug, a conjugate between TP and carboxymethyl chitosan (CC). The CCTP conjugate contained 4∼ wt % TP and displayed excellent aqueous solubility (5 mg/mL) as compared to the native TP (17 μg/mL). *In vitro* cytotoxicity of CCTP conjugate was evaluated by CCK8 assay against human pancreatic cancer (PC) cell lines, showing comparable the half maximal inhibitory concentration (IC_50_) values to the parent TP. In a mouse model of PC (BxPC-3), the CCTP conjugate administered orally (at dose levels as low as 0.2 mg TP equivalent/kg) showed comparable efficacy in reducing or eliminating xenograft tumor to the same dose of TP, but exhibited much lower subacute toxicity as seen in body weight loss and hematological toxicity.

## 1. Introduction

Pancreatic cancer (PC) is an aggressive and highly lethal human malignancy with an all-stage 5-year survival rate of <5%, which accounts for 2% of all cancers but 5–6% of cancer deaths worldwide (McGuigan et al., 2018). Chemoresistance is a key factor preventing response to therapies in PC, which can help tumor elude cell death under various survival mechanisms (Wang et al., 2016). Gemcitabine as the first-line drug has been used to treat PC; however, the insensitivity due to singleness of therapeutic target limits its clinical effect. In addition, gemcitabine confers a median survival advantage of just 6 months, a slight improvement of 1 month over its predecessor 5-fluorouracil (5-FU) (Giri et al., 2017; Chugh et al., 2012). Hence, there are still urgent needs to develop a novel chemotherapeutic drug to overcome the drug resistance in PC.

Some reports have indicated that triptolide (TP), a diterpenoid triepoxide isolated from the *Tripterygium wilfordii* Hook F (TWHF), is superiorly effective against PC cells compared to gemcitabine and taxanes *in vitro* as well as *in vivo*.4 (Li et al., 2016; Zhu et al., 2012; Herreros-Villanueva et al., 2012). TP can induce cell death in PC by multiple pathways, such as autophagy and apoptosis (Mujumdar et al., 2010; Phillips et al., 2007). Therefore, TP is considered to have great promise of its clinical translation. However, its potential clinical use is restricted to the poor solubility (0.017 mg/mL) in water and toxic effects (LD_50_, 0.8 mg/kg, mouse) toward healthy tissues, such as liver, renal, hematopoietic and reproductive systems (Shamon et al., 1997).

To address these issues and toward clinically safe application of TP, we developed a highly water-soluble TP conjugate. Natural low molecular weight polysaccharide, carboxymethyl chitosan (CC), was first utilized as TP carriers to form 14-succinate triptolide-carboxymethyl chitosan (CCTP) conjugate. Here, we present the preclinical assessment of CCTP in tumor cells and animal models of PC. The *in vitro* efficacy of CCTP was compared with that of its parent TP. Subsequently, the *in vivo* toxicity and antitumor efficacy of CCTP was evaluated in animal models, including normal mice and human xenograft models of PC. We observed that CCTP effectively decreased tumor burden and tumor-associated morbidity, as well as increased overall survival in xenograft animal models of PC. CCTP was more effective than its parent TP in reducing cytotoxicity in v*itro* and *in vivo.* The results demonstrated the antitumor efficacy and safety of CCTP therapeutics as well as its potential clinical applications as a chemotherapeutic drug.

## 2. Materials and methods

### 2.1. Materials

TP was purchased from Xi’an Haoxuan Biotechnology Co. Ltd. (Shanxi, China). CC was obtained from Aladdin (Beijing, China) and was further purified by dialysis. Hyclone fetal bovine serum, bovine serum albumin (BSA) and Dulbecco’s modified Eagle’s medium (DMEM) were supplied by Gibco Co. Ltd. (USA). Freund’s complete adjuvant was from Sigma (USA). Human pancreatic cancer cells (BxPC-3) and hepatic cells (HL7702) were from the American Type Culture Collection (VA, USA). All other reagents and solvents were purchased from commercial suppliers with the analytical grade. BALB/c mice (20-22 g) were purchased from the Huaxing laboratory animal farm (Production license NO: 20190002, Zhengzhou, China). The animals were allowed to acclimatize for seven days in environmentally controlled quarters (25 ± 1 °C, 12 h light-dark cycle), provided with water and food. All animal studies were performed under a protocol approved by the Institute’s Animal Care and Use Committee.

### 2.2. Synthesis and characterizations of CCTP conjugate

A 2′-hemisuccinate ester (TP-Suc) of TP was prepared by a previously reported method with some modification (Lee et al., 2009). Briefly, TP (360 mg, 1 mmol), succinic anhydride (400 mg, 4 mmol), and DMAP (24 mg, 0.2 mmol; 4-dimethylaminopyridine) were dissolved in pyridine (10 mL). The mixture was stirred for 24 h under nitrogen purging at room temperature. After removal of the solvent under vacuum, the residue was purified by silica gel column chromatography.

TP-Suc (50 mg, 0.11 mmol) dissolved in anhydrous DMSO (10 mL) was activated by NHS (50 mg, 4 eq; N-hydroxy succinimide) and EDC (45 mg, 2 eq; 1-ethyl-3-(3-dimethylaminopropyl) carbodiimide hydrochloride) for 3 h at room temperature. Subsequently, the mixture was added to CC (300 mg, 15-20 KDa) dissolved in mixed solutions of DMSO (20 mL) and borate buffer (35 mL, pH10). After stirring for 24 h at room temperature, the mixture was diluted by cold distilled water (2×) and dialyzed against distilled water using a dialysis membrane (cut off: 3500 Da), and then CCTP was lyophilized into white solid. The products were characterized using IR spectrum. CCTP was analyzed by UV-vis spectroscopy to obtain the TP weight percentage at a wavelength of 218 nm.

### 2.3. Properties studies

For the solubility studies, CCTP (or TP) (1 0 –15 mg) was added in 1.0 mL of redistilled water. The mixture was vortexed for 5 min, sonicated for 2 min, and centrifuged at 14,000 rpm for 10 min. The supernatant was saturated solution, and was quantified by UV spectrophotometer.

*In vitro* drug release from CCTP was tested in cell culture medium (10% FBS), plasma, simulated gastric fluid (SGF; 0.32% pepsin, pH 1.2), and simulated intestinal fluid (SIF; 1% pancreatin, pH 7.5) at 37 °C for 12 h. CCTP (40 mg) was dissolved in 10 mL of each solution. And then, 200 μL of samples were taken at predetermined times. The released free TP was subsequently extracted by using 1 mL of dichloromethane and was quantified by HPLC on a C18 column (4.6 × 250 mm, Agilent) with acetonitrile/water (33:67, 1 mL/min).

### 2.4. *In vitro* cytotoxicity study

We compared the cytotoxicity of CCTP with that of parent TP against human pancreatic cancer cell (BxPC-3) and hepatic cell (HL7702). The cells were respectively incubated for 48 h in the culture medium containing TP (8-1024 nmol/L in DMEM with 1‰ DMSO) or CCTP (at equivalent concentration of TP in DMEM). After incubation, the viability of cells was determined by adding 10 μL CCK-8 into each well. The optical density of the solution was measured by a microplate reader (Thermo, USA) under 450 nm absorbance values. The values (the half maximal inhibitory concentration (IC_50_)) of 50% inhibitory concentration of TP and CCTP on cells were calculated by GraphPad Prism7 software.

### 2.5. Apoptosis detected by flow cytometry

Bxpc3 cells were incubated with TP (55.5-222 nmol/L) or CCTP (at equivalent concentration of TP). After 24 h incubation, cells were centrifuged and washed with PBS for three times, incubated in 500 μL of binding buffer with 10 μL of PI and 5 μL of Annexin V-FITC for 5 minutes. The apoptosis rate of the different groups was detected by Cytoflex (Beckman Coulter, USA).

### 2.6. *In vivo* toxicity

Toxicity of CCTP was evaluated using normal BALB/c mice. The mice were divided into five groups randomly (*n* = 9) and were administered TP (0.2 mg/kg), CCTP (0.2, 0.4, 0.8 mg TP equivalent/kg), and saline (Control), respectively. The mice were administrated orally (p.o.) once every 2 days for seven times. The body weight was examined every day for 14 days, and the blood samples were collected from eyes on the 14th day. Hematological parameters, including number counts of white blood cells (WBC), red blood cells (RBC), and neutrophils, were measured. The renal function indexes (blood urea nitrogen (BUN) and creatinine (Crea)) and the hepatic function indexes (serum alanine aminotransferase (ALT) and aspartate aminotransferase (AST)) were detected by Beckman CX5 Automated analyzer.

### 2.7. *In vivo* antitumor effect

Human pancreatic cancer cells (BxPC-3 cell, 1 × 107, 100 μL) were implanted subcutaneously into two BALB/c nude mice. When solid tumor volumes reached 300–400 mm^3^, tumors were dissected and cut into 10 mm^3^ pieces, which were subsequently subcutaneously re-implanted into right flanks of other mice. When the tumor node had reached 100 mm^3^, mice were randomized into five treatment groups (*n* = 9) and were administrated orally with either saline, TP [0.2 mg/kg (every two days)] or CCTP [equaling to 0.2, 0.4, and 0.8 mg/kg of TP (every two days)]. Tumor volume and body weight were monitored every two days. Tumor volume (mm^3^) was measured using calipers and calculated using the equation (Legends × Width^2^/2). Subsequently, mice were sacrificed in accordance to institutional guidelines, and the kidneys, livers, and tumors were harvested and stored at −80 °C.

### 2.8. Histological examination

The kidneys, livers, and tumors of each group were fixed with 4% formalin. After decalcification with 10% EDTA, dehydration with gradient alcohol, paraffin embedding, and pathological section, hematoxylin and eosin (HE) staining was performed in the paraffin slices for histological examination.

### 2.9. Data analysis

SPSS 17.0 statistical package was used for the analysis. All data were expressed as the mean ± SD. Statistical analysis was performed using the two-tailed Student’s *t*-test. A value of *p* < 0.05 was considered statistically significant.

## 3. Results and discussion

### 3.1. Synthesis and characterization of CCTP

**Figure 1.**
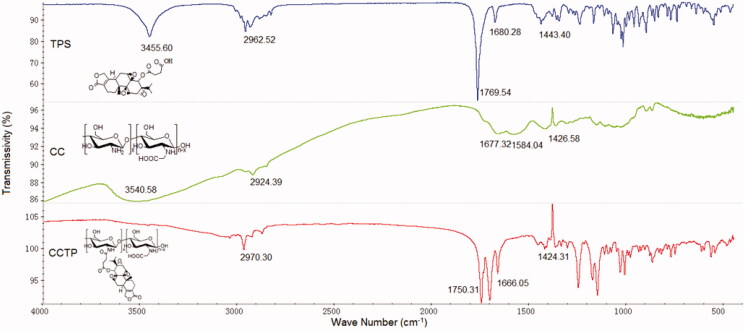
IR spectra of CCTP conjugate.

CCTP conjugate was synthesized through covalent attachment from TP and CC with a succinate as linker that can be cleaved to release a parent TP under physiological conditions (Lee et al., 2009; Safavy et al., 2004). CCTP was characterized by IR spectrum, which showed corresponding peaks to both TP and CC (Figure 1). The UV spectrum of CCTP revealed that the conjugate contained 4∼ wt % TP and had much greater water solubility (5 mg/mL) than the native TP (∼17 μg/mL). The results indicated that the conjugation of TP to CC effectively improved water solubility of TP.

**Figure 2.**
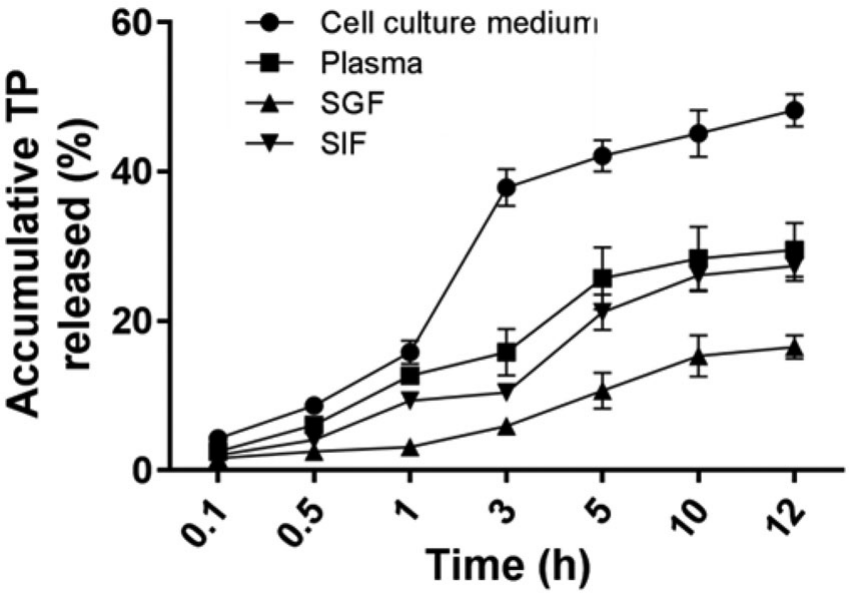
The controlled release manner of CCTP with times upon incubation in cell culture medium (10% FBS), mice plasma, SGF (simulated gastric fluid, pH 1.2), and SIF (simulated intestinal fluid, pH 7.5); Data presented as means ± SD, *n* = 3.

*In vitro* drug release from the conjugate analysis described the stabilities and controlled release ability of TP from CCTP in cell culture medium, mouse serum, SGF and SIF, respectively (Figure 2). When HPLC was used to detect the free TP, we found that the retention time of the free TP released from the conjugate was consistent with that of the parent TP, which suggested the ester bonds on linking group of CCTP were cleaved by some kind of ester hydrolytic catalyst in those solutions. The similar extent of TP release from the conjugate was observed within 12 h for SIF (27.4%) and plasma (29.5%), respectively. Only ∼5.9% of TP was released from CCTP at 3 h post-incubation in SGF, whereas great amounts of drug release were observed in cell culture medium (∼38%). The results indicated that CCTP conjugate is relatively stable in SGF with gastric acid and propepsin, while it is very easy to hydrolyze in cell culture medium with complex components, including serum, amino acids, vitamins, and so on. Considering the transit time in stomach (about 3 h), TP release in SGF would seem insubstantial. Overall, the drug release analysis indicates clearly that most of TP release does not take place under gastric environment, but may mainly happen during or after uptake by the intestine and transport to the blood capillary.

### 3.2. *In vitro* cytotoxicity of CCTP

**Figure 3.**
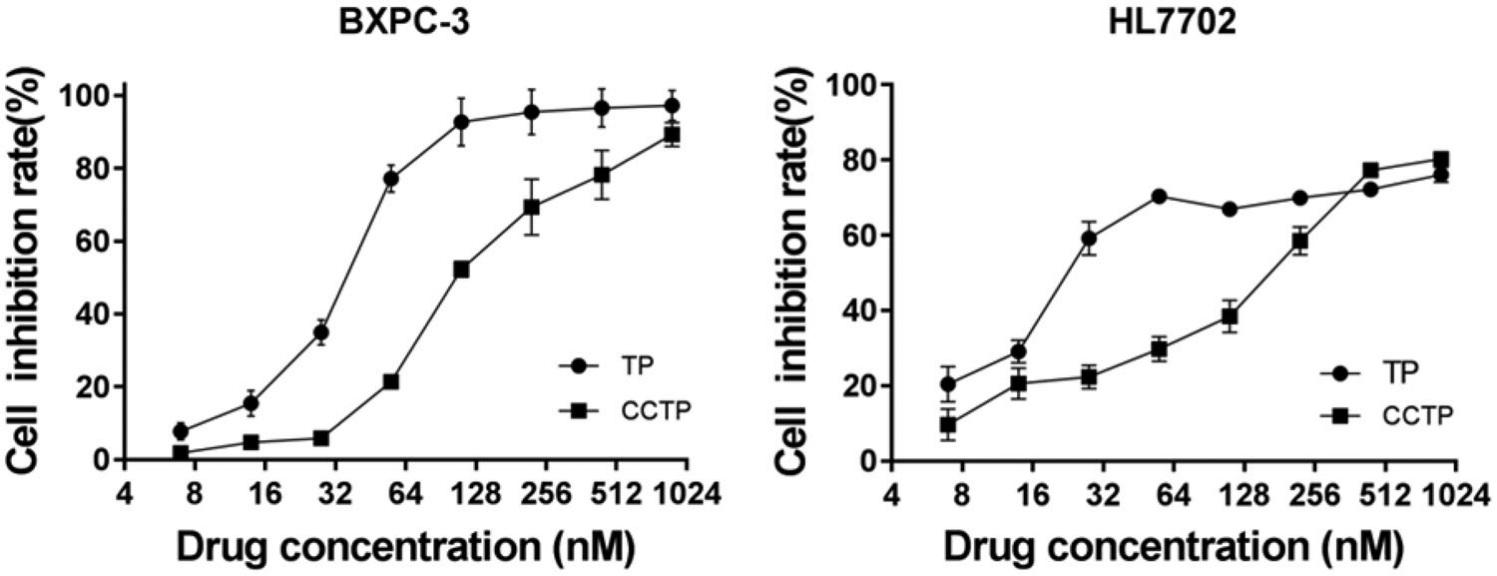
The *in vitro* cytotoxicity of free TP or CCTP on BxPC-3 cells and HL7702 cells.

Figure 3 shows the *in vitro* cytotoxicity of TP and CCTP against human pancreatic cancer cell (BxPC-3) and hepatic cell (HL7702), respectively. The IC_50_ values of TP and CCTP are 38.22 ± 0.52 vs 110.74 ± 0.35 nmol/L for BxPC-3 and 24.04 ± 0.71 vs 154.27 ± 0.9 nmol/L for HL7702, respectively. A slight activity loss for BxPC-3 cell by less than 3 fold and a cytotoxicity decrease for hepatic cell (HL7702) by more than 6 fold were observed with CCTP conjugate form, which could be accounted for the gradual release of TP from the conjugate through cleavage of the succinate linker between CC and TP. In addition, the cell inhibition rates of TP and CCTP (380 nmol/L of TP) were the same in HL7702 cell groups, which indicated that the TP were almost released from the CCTP. These data suggest that CCTP can reduce toxicity and other side effects in inhibition of hepatocyte proliferation.

### 3.3. CCTP decreased TP-induced apoptosis

**Figure 4.**
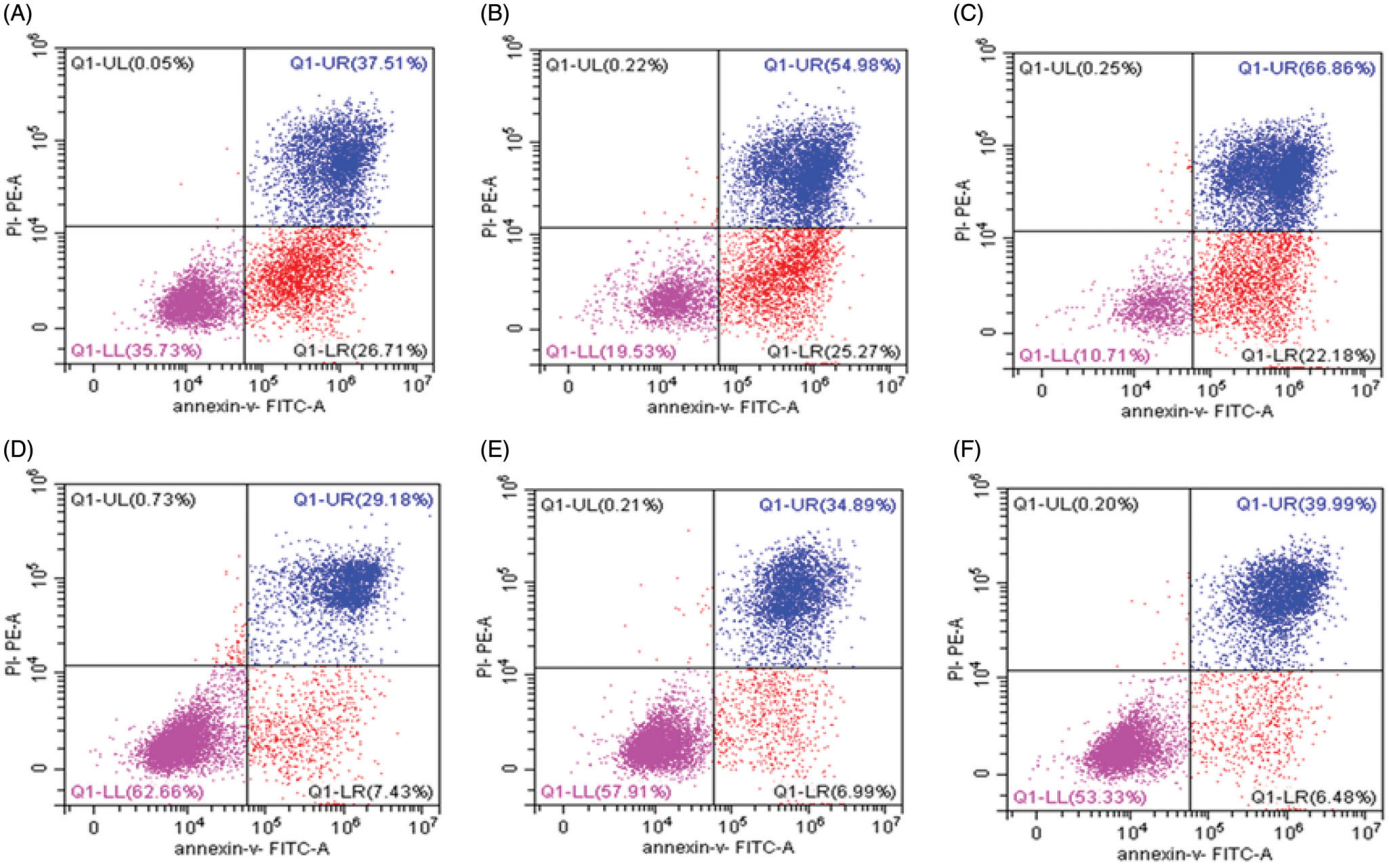
The BxPC-3 cell apoptosis induced by TP (A-C) and CCTP (D-F) detected by flow cytometric method. The concentrations of TP or CCTP (equal to TP concentration) are 55.5 nmol/L (A, D), 111 nmol/L (B, E), 222 nmol/L (C, F), respectively.

The effect of the drug delivery system on TP-induced apoptosis in human PC cells was tested by a flow cytometric method, as shown in Figure 4. The *in vitro* experiments showed that the early apoptosis rates gradually decreased from 26.7%, 25.3% to 22.2% and the late-stage apoptosis rates increased from 37.5%, 54.9%, to 66.9% after treatment with 55.5 nmol/L, 111 nmol/L, and 222 nmol/L of TP, respectively, for 24 h (Figure 4(A–C)). This suggests that TP induces BxPC-3 cell apoptosis and causes a significant increase in cell apoptosis with a concentration-dependent manner. After incubation with CCTP (at equivalent TP concentrations) for 24 h, the apoptosis rates of PC cells were decreased from 7.43%, 7.0% to 6.5% (at early apoptosis stage), and increased from 29.2%, 34.9% to 40.0% (at late apoptosis stage) (Figure 4(D–F)). The flow cytometry data revealed that under the same conditions the apoptotic cells were less in CCTP group than those in TP group. The results indicated that the drug delivery system had delay effects on TP-induced cell apoptosis.

### 3.4. *In vivo* toxicity of CCTP

**Figure 5.**
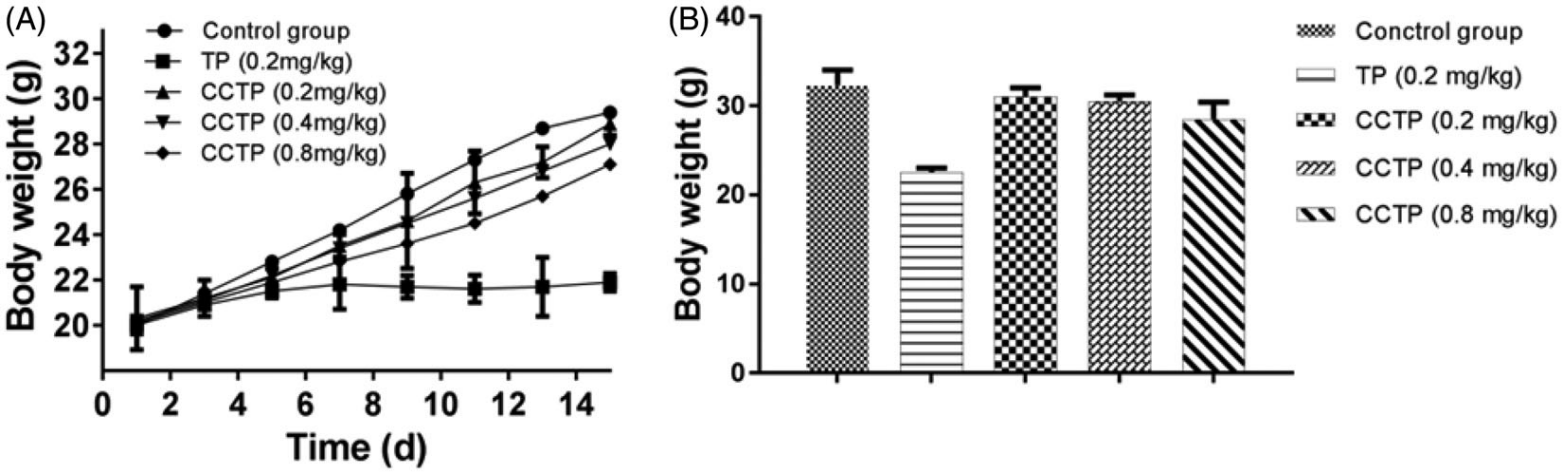
*In vivo* toxicity of CCTP: (A) Monitoring of body weight; (B) Body weight at the end of the experiment during administrations in normal BALB/c mice with saline, TP (0.2 mg/kg, p.o.) and CCTP (0.2 mg TP equivalent/kg, p.o.). Data presented as means ± SD, *n* = 9.

*In vivo* toxicities of TP and CCTP were compared by using normal BALB/c mice that were evaluated by monitoring body weight change and hematological toxicity (Figure 5). Previous experiments showed that the minimum lethal dose of mice was about 0.5 mg TP/kg. Here, a safe dose of free TP of 0.2 mg/kg was adopted for mice in toxicity assay. There was no difference in body weight between CCTP and control group, whereas TP group showed diminution of body weight of about 30.3% during oral administration period (Figure 5). In addition, TP group showed severe hematological toxicity in terms of a decrease in the number of neutrophil over 45%. In contrast, CCTP group exhibited a slight decrease of red blood cells, white blood cells and neutrophil (Table 1). We speculate that the significant toxicity attenuation should be attributed to the gradual or sustained release of TP from CCTP conjugate into blood stream after oral administration.

**Table 1.** Biochemical parameters of TP- and CCTP-treated animals.

	RBC	WBC	Neutropenia
Control group	100 ± 2.28	100 ± 1.68	100 ± 3.98
TP	94 ± 4.76	80 ± 1.12	55 ± 3.82
CCTP	103 ± 3.55	98 ± 2.32	87 ± 2.93

Increased serum BUN and Crea concentrations indicated the kidney dysfunction (Table 2). Significant increase of BUN indicated the histomorphological changes including dilation of kidney proximal tubules (Liu et al., 2008). Here, high dose of CCTP and TP groups had higher BUN and Crea than the control group (*p* < .05), while middle dose of CCTP showed moderate effects on BUN and Crea. There were no significant differences in BUN and Crea between low dose of CCTP and control groups (*p* < .05). Compared to the control group, TP group showed great increase of both AST and ALT, indicating the occurrence of liver injury. For low and middle doses of CCTP, both groups showed no significant elevation (*p* < .05). However, by increasing the dose to 0.8 mg TP equivalent/kg, the CCTP group showed that both ALT and AST were elevated to some extent, compared to the control group. The results demonstrated that the TP treatment was associated with nephrotoxicity and hepatotoxicity. In addition, as the dose increase, CCTP showed increasing toxicity associated with kidney and liver. The CCTP group underwent mild elevation in BUN, Crea, AST and ALT levels, indicating that the incidence of CCTP induced kidney and liver toxicity is very low.

**Table 2.** Biochemical parameters of TP and CCTP treated model animals ($\bar{\text{x}}$± s, *n* = 9).

	BUN（mmol/L）	CRE(μmol/L)	AST(IU/L)	ALT(IU/L)
Control group	24.35 ± 2.98	33.58 ± 6.32	45.17 ± 8.96	33.36 ± 4.65
TP (0.2 mg/kg)	51.32 ± 9.21*	71.34 ± 12.22*	148.22 ± 27.56*	78.55 ± 12.23*
CCTP (0.2 mg/kg)	24.46 ± 2.63**	33.37 ± 7.81^#^	48.38 ± 18.23**	33.54 ± 8.99**
CCTP (0.4 mg/kg)	25.32 ± 4.81 ^##^	37.55 ± 11.32^##^	52.34 ± 17.85^##^	35.56 ± 10.67^* ##^
CCTP (0.8 mg/kg)	38.82 ± 4.96 ^##^	49.45 ± 10.15^##^	98.12 ± 19.78^##^	65.13 ± 13.45 ^##^

### 3.5. Antitumor effect of CCTP in xenograft models

**Figure 6.**
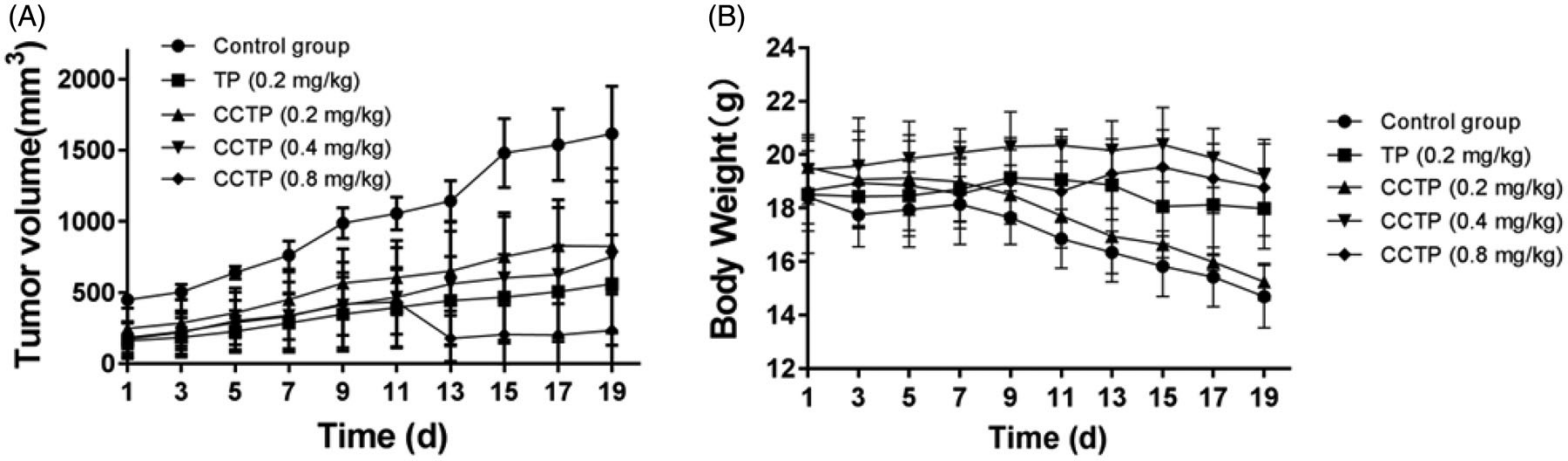
Efficacy and tolerability of TP and CCTP in a mouse model bearing human pancreatic cancer with oral administration every two days. (A) BxPC-3 solid tumor volume over time; (B) mouse body weight over time.

The antitumor efficacy of CCTP in human PC xenograft models were evaluated by monitoring tumor growth and body weight change (Figure 6). CCTP (0.8 mg TP equivalent/kg) substantially inhibited the tumor growth of 85.49% in comparison with control group on day 19, which is better than antitumor efficacy in TP (0.2 mg/kg), exhibiting tumor growth inhibition of 65.35% (Figure 6(A)). CCTP (0.4 mg TP equivalent/kg) also showed the tumor growth inhibition of 53.51% (*p* < .05), whereas the same antitumor activity was observed with CCTP (0.2 mg TP equivalent/kg). The body weight change may reflect the complex effects of tumor progression and drug toxicity (Figure 6(B)). The control group showed a marked weight loss for up to 19 days, indicating the tumor progression was the major factor of weight loss. The low dose of CCTP (0.2 mg TP equivalent/kg) group showed a slight difference of weight loss as compared to the control group (*p* > .05). In contrast, the medium and high doses of CCTP groups exhibited a slight weight increase, indicating the toxicity and antitumor efficacy of the conjugate were correlated with the dosage. However, TP (0.2 mg/kg) group kept the body weight relatively constant when compared to the control group. The results indicate that CCTP is comparably effective in inhibiting tumor growth but is much less toxic compared to TP. We think that the controlled release of CCTP conjugate maintains a long-term equilibrium concentration of TP *in vivo*, which can enhance the efficacy of CCTP and the biological safety.

**Figure 7.**
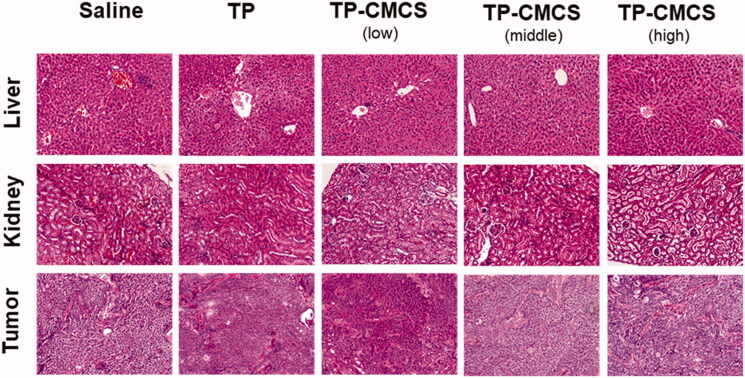
Histological staining of organs and tumor following oral administration of TP formulations (×200).

Figure 7 represents an optical micrograph of liver, kidneys, and tumor tissue of mice treated with saline, TP, and CCTP, respectively. In the saline group, tumors were composed of dense cells with large and regular nuclei. The TP group showed a lot of cancer cell necrosis, indicating TP with good effect for inhibiting cancer cells. The group treated with CCTP displayed a wide range of cancer cell necrosis and a lot of cancer cells undergo apoptosis, scattered focal cancer cell nests arranged in gland tube-like. Especially the high dose CCTP group exhibited superior anticancer efficacy compared to TP group, which should be attributed to CC polymer provide CCTP an approach to achieve controlled release of TP and high water solubility. Histopathological changes in liver and kidneys were discovered in TP and CCTP groups. Fatty degeneration in the hepatocytes and obvious kidney proximal tubular dilation were observed in mice after oral administration of TP (Figure 7). However, in the CCTP groups at the low, medium, and high dose, no apparent changes were found, which is also consistent with the hematological toxicity results.

## 4. Conclusions

A CC prodrug of the antitumor agent TP was prepared from the natural product and showed excellent water solubility (5 mg/mL). CCTP conjugate effectively inhibited tumor growth with an efficacy comparable to TP even at the same dose level, but with much lower toxicity, indicating high potential for further clinical steps. The strong antitumor activity and lower toxicity of CCTP after oral administration may be attributable to its greater water solubility and controlled release. As a consequence, we suggest CC-based conjugate system may be a useful tool for oral delivery of other poorly water-soluble drugs.
